# Strategies for enhancing CAR T cell expansion and persistence in HIV infection

**DOI:** 10.3389/fimmu.2023.1253395

**Published:** 2023-08-21

**Authors:** Frederik Holm Rothemejer, Nanna Pi Lauritsen, Ole Schmeltz Søgaard, Martin Tolstrup

**Affiliations:** ^1^ Department of Clinical Medicine, Aarhus University, Aarhus, Denmark; ^2^ Department of Infectious Diseases, Aarhus University Hospital, Aarhus, Denmark

**Keywords:** chimeric antigen receptor, HIV, persistence, expansion, TCR-engagement, CAR T cell persistence

## Abstract

Chimeric Antigen Receptor (CAR) T cell therapies are tremendously successful in hematological malignancies and show great promise as treatment and curative strategy for HIV. A major determinant for effective CAR T cell therapy is the persistence of CAR T cells. Particularly, antigen density and target cell abundance are crucial for the engagement, engraftment, and persistence of CAR T cells. The success of HIV-specific CAR T cells is challenged by limited antigen due to low cell surface expression of viral proteins and the scarcity of chronically infected cells during antiretroviral therapy. Several strategies have been explored to increase the efficacy of CAR T cells by enhancing expansion and persistence of the engineered cells. This review highlights the challenges of designing CAR T cells against HIV and other chronic viral infections. We also discuss potential strategies to enhance CAR T cell expansion and persistence in the setting of low antigen exposure.

## 1 Introduction

While Chimeric antigen receptor (CAR) T cell therapy has emerged as a potent treatment against several hematological malignancies (1–5), it was initially developed as a strategy for durable control of HIV. Although the concept was proved safe and engineered cells could be detected 10 years after infusion, results from early clinical trials did not show any impact of HIV-specific CAR T cell therapy on the latent viral reservoir (6–12). Subsequent improvements to the design of the CAR, specifically inclusion of an intracellular co-stimulatory signaling domain along with the CD3ζ domain, have proved vital to increase the efficacy of CAR T cells (13, 14). After the clinical success of CAR T cells targeting CD19 or BCMA in B cell malignancies, the field has expanded substantially and interest in CAR T cells as treatment for other diseases has been renewed. CAR T cells are now being explored against a range of diverse conditions, including solid tumors, rheumatological disease and chronic viral infections (15–19). However, the immunological landscape varies in these conditions giving rise to new challenges for effective CAR T cell therapy. Following the initial trials with HIV-specific CAR T cells and the considerable advances to the CAR design achieved in hematological indications, there is a renewed interest in using CAR T cells as a curative approach for HIV. Several HIV-specific CAR T cells are now being evaluated in published (20) and ongoing clinical trials (NCT04648046 and NCT03617198). HIV-specific CAR constructs are typically based on the CD4 ectodomain or single-chain variable fragments (scFv) derived from broadly neutralizing anti-Envelope (Env) antibodies. Several studies have further engineered the CAR T cells to become resistant to HIV infection through HIV co-receptor disruption or expression of entry inhibitors (20–28). However, one key obstacle to effective anti-HIV CAR T cell therapy is the persistence of the engineered cells *in vivo*.

**Figure 1:**
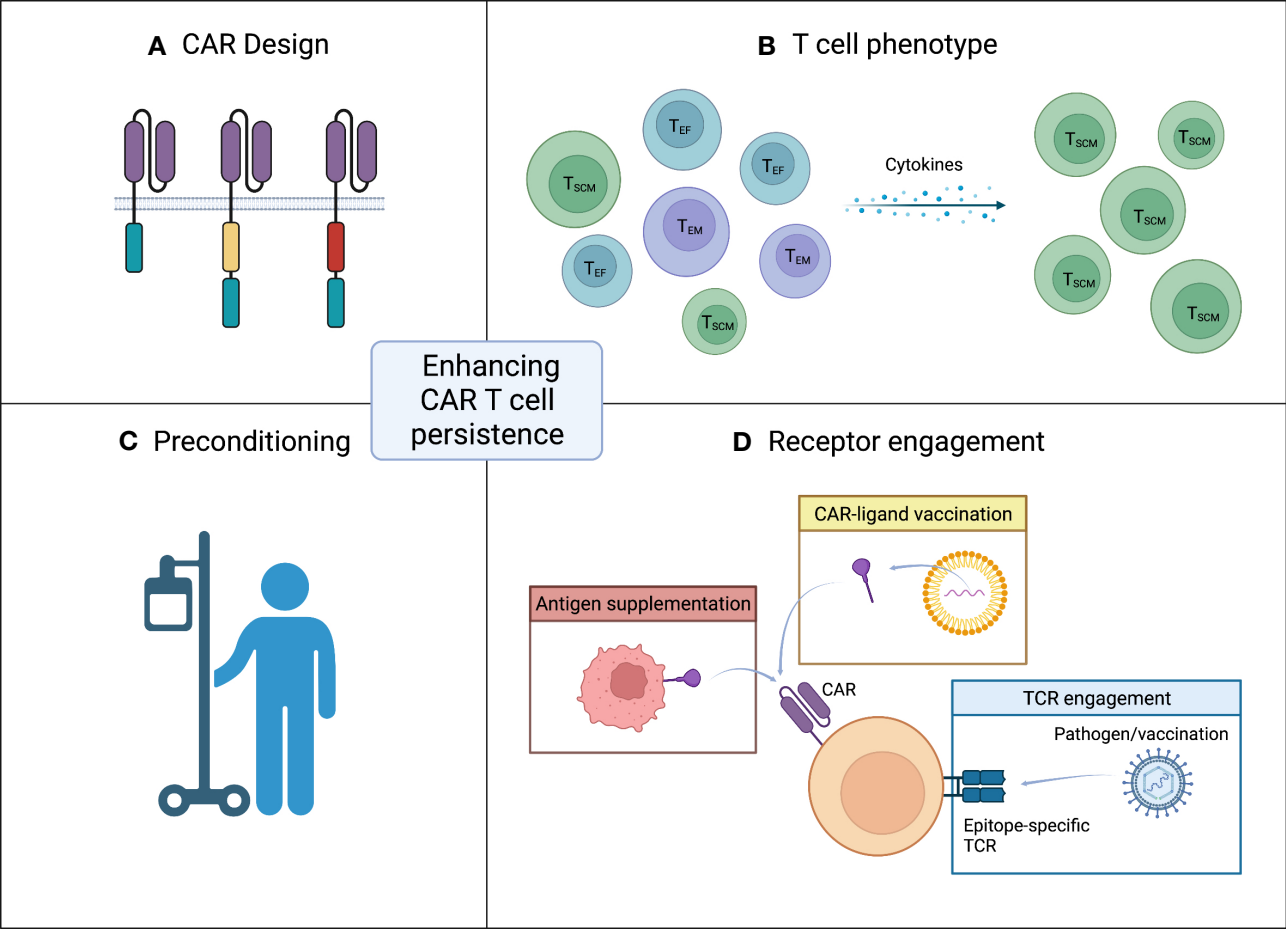
Strategies for enhancing CAR T cell persistence. **(A)** CAR design. The design of the CAR with inclusion and choice of co-stimulatory domain has great impact on persistence. **(B)** T cell phenotype. A less differentiated memory like phenotype of infused CAR T cells is favorable. **(C)** Preconditioning. Effective lymphodepleting preconditioning prior to CAR T cell infusion can increase CAR T cell persistence. **(D)** Receptor engagement. Target antigen encounter is crucial for the CAR T cells to persist after infusion. In the setting of low antigen exposure CAR engagement can be ensured by either vaccination (yellow box) or antigen supplementation (red box). Another approach to accommodate low level target antigen is engagement of the native TCR (blue box). Figure created with BioRender.com.

Persistence of CAR T cells has been identified as a major determinant for treatment response (3, 4, 29). Several studies have demonstrated that the antigen density and subsequent CAR engagement with the antigen are crucial for CAR efficacy and engraftment (30–32). During ART-suppressed chronic HIV infection, infected cells typically have low levels of HIV antigen on their surface and in addition, latently infected cells are rare (33–36). These properties are in stark contrast to the high tumor burden and high antigen density observed in B cell malignancies. This gives rise to a barrier in ensuring persistence of CAR T cells directed towards HIV. In this mini-review, we address strategies to enhance the expansion and persistence of CAR T cells that are required for effective HIV-specific CAR T cell therapies in chronic HIV infection (**Figure 1**).

## 2 Importance of persistence to treatment response

A major determinant for treatment response to CAR T cell therapies in B cell malignancies is the expansion and persistence of the CAR T cells (3, 4, 29). Several trials have demonstrated that the presence of CAR T cells in circulation months after infusion is associated with long-term treatment response (3, 29, 37). Both the cumulative CAR T cell expansion and peak numbers of CAR T cells after the initial expansion phase have been found to be significantly higher for patients responding to anti-CD19 CAR T cell therapy compared to non-responders (29, 38, 39). Additionally, patients with long-term persistence of CAR T cells have a greater initial peak of CAR T cell levels than patients without persistence of the CAR T cells (29, 37). Due to the essential role of target antigen density, patients with higher pre-treatment tumor burden and higher target antigen density have been shown to have a greater peak and accompanying expansion of the CAR T cells (38, 40). However, high pre-treatment tumor burden is generally associated with worse outcome. These studies demonstrate that the ability of the infused CAR T cells to expand and persist are crucial for the treatment success.

In order for CAR T cells to expand, the CAR or T cell receptor (TCR) has to engage with the specific antigen. A native TCR is 100-fold more sensitive to antigen than a CAR where it is estimated that a threshold of 200 target antigen molecules per cell is required for lytic activity of the CAR T cells and 10-fold higher for cytokine production (31, 41). The requirement for high antigen exposure poses a challenge for effective CAR T cell therapy in diseases where the antigen density is low, such as HIV. The sole surface antigen available for immune recognition of HIV-infected cells is the Env composed of the glycoprotein subunits gp120 and gp41. However, Env is expressed at very low levels both on virions and on the surface of infected cells (33, 35). Additionally, during ART suppression the Env expression decrease and latently infected cells become exceedingly rare (36). Combined, the low antigen density of HIV Env and the scarcity of latently infected cells challenge the expansion and persistence of anti-HIV CAR T cells due to insufficient CAR engagement.

## 3 Strategies for enhancing CAR T cell persistence

### 3.1 Impact of CAR design on persistence

In the first generation of CARs, signaling through CD3ζ alone did not induce an adequate treatment response. In subsequent generations of CARs two or more co-stimulatory signaling domains have been incorporated with greatly increased efficacy (11, 42) (**Figure 1A**). The most common design is the second generation CARs composed of an extracellular scFv fused to the intracellular signaling domains of either CD28 or 4-1BB along with CD3ζ (43). Several studies have compared the impact on proliferative capacity and persistence of second generation CARs using either CD28 or 4-1BB co-stimulatory signaling. Pre-clinical studies typically favor 4-1BB CARs although differences in efficacy are not as evident in clinical trials where other variations in CAR design and choice of delivery vector might also affect the differences observed in CAR T cell function (43–46).

Engagement of CD28-based CARs rapidly activates T cell effector functions relying on glycolytic metabolism inducing an effector phenotype. Engagement of 4-1BB-based CARs promotes a less differentiated memory phenotype of the T cell relying on oxidative metabolism and mitochondrial biogenesis. This leads to an enhanced persistence of the 4-1BB-based CAR T cells (45, 47–50). Additionally, the increased persistence of CAR T cells incorporating 4-1BB is further hypothesized to be due to a slower initial tumor clearance compared to CD28 CAR T cells. The slower tumor clearance results in longer antigenic stimulus of the 4-1BB-incorporating CAR T cells (51).

Furthermore, CD28-based CARs usually comes with an increased incidence of cytokine release syndrome, neurotoxicity and with higher rates of severe adverse events (13). For HIV-specific CAR T cells, Maldini et al. explored the choice of co-stimulatory domain in the CAR design in a murine study where 4-1BB-based CARs showed a proliferative advantage (23). However, T cells transduced with both a CD28 and a 4-1BB-based CAR, showed superior proliferation and mitigation of CD4+ T cell loss which was not observed for third generation CARs composed of both CD28 and 4-1BB in the same construct (23). Similarly, Leibman et al. showed in mice that 4-1BB-based CAR T cells reach greater numbers in both HIV-infected and uninfected humanized mice (22).

Further modifications of the CAR architecture could potentiate the expansion and persistence of CAR T cells in the setting of low-level antigen exposure (**Figure 1A**). The scFv linker, extracellular hinge/spacer and transmembrane domain all have profound impact on the functionality of the CAR T cells (52–56). Similarly, mutations in the intracellular signaling domains to avert tonic signaling have led to increased persistence and antitumor efficacy (57, 58). Several other co-stimulatory signaling domains and combinations thereof have been evaluated along with co-expression of cytokines or gene circuits with promising results. These additional CAR modifications have been reviewed extensively elsewhere (59–61).

### 3.2 Producing CAR T cells of less differentiated memory phenotypes

The phenotype of engineered CAR T cells greatly influence the potency and proliferative capacity of the cells (**Figure 1B**). CAR T cells produced from less differentiated memory phenotypes, e.g., naïve and stem cell memory T cells, compared to either bulk or more differentiated phenotypes have higher antileukemic potency and greater proliferation in pre-clinical trials (62–67). Analyses of responders to anti-CD19 CAR T cell therapy in clinical trials further reveal that a less differentiated memory phenotype positively impact the efficacy and persistence of the CAR T cells (68–71). CAR T cells with a specific phenotype can be attained by either enrichment of cells with the desired phenotype or by supplementing the cell culture medium with specific cytokines known to modulate the phenotype of T cells (72–76). By expanding the CAR T cells in the presence of a combination of IL-7, IL-12, IL-15 and IL-21, cells exhibiting a less differentiated phenotype are retained resulting in a more durable antitumor response and greater persistence (72–74). Alternatively, anti-HIV CAR T cells derived from hematopoietic stem and progenitor cells (HSPC) have shown long-term persistence in NHPs (77–80). The regenerative nature of HSPCs makes these a promising choice for producing CAR T cells with long-term persistence although obtaining sufficient HSPCs from adult patients will be limiting with current technologies.

### 3.3 Preconditioning prior to CAR T cell infusion

Several clinical trials have shown enhanced persistence of CAR T cells when patients are preconditioned with effective lymphodepleting most often using cyclophosphamide and fludarabine (40, 46, 81–83) (**Figure 1C**). Lymphodepleting preconditioning could have an additional beneficial effect when HIV-specific CAR T cells are used as a curative strategy as a means of lowering the size of the latent viral reservoir. Published cases of HIV-1 cure after *CCR5Δ32/Δ32* HSPC transplantation have all received lymphodepleting preconditioning which is known to drastically reduce the size of the viral reservoir by eliminating host CD4+ T cells (84–91). However, careful consideration and optimization of the pre-CAR T cell lymphodepletion regimens to lessen adverse effects are required before use in otherwise healthy people with HIV.

### 3.4 Receptor engagement

Due to the crucial role of target antigen density for CAR T cell persistence, multiple strategies for ensuring receptor engagement have been employed. CAR engagement can be facilitated by increasing the level of available antigen. Increased HIV antigen can be achieved through the use of latency reversing agents (LRAs) which is a thoroughly studied concept in HIV cure trials. Several LRAs have been evaluated in clinical trials where they are safe and can induce viral reactivation in ART-suppressed participants (92–96). Target antigen density and subsequent CAR engagement can also be increased through vaccination or antigen supplementation (**Figure 1D**). An alternative approach is to engage the native T cell receptor (**Figure 1D**). In this section, these concepts will be discussed.

#### 3.4.1 CAR engagement by vaccination

In indications with low CAR-specific antigen densities, sufficient antigen levels can be achieved through vaccination with the CAR-specific antigen (**Figure 1D**). CAR engagement through vaccination has the advantage of having the CAR-specific antigen presented by antigen presenting cells (APC) in lymphoid tissues and thus providing additional immunostimulatory signaling. Ma and colleagues (97) evaluated an amphiphile CAR T cell ligand that traffics to draining lymph nodes upon injection where it decorates the surface of APCs. Mice receiving a low dose of 5 x 10^4^ CAR T cells followed by vaccination with the amphiphile CAR T cell ligand had significantly more CAR T cells in the peripheral blood 14 days after vaccination than mice receiving a 20-fold higher dose of 10 x 10^6^ CAR T cells (97). Likewise, Reinhard and colleagues (98) developed a lipid nanoparticle (LNP) RNA vaccine encoding the CAR-specific antigen. Similar to Ma et al, mice receiving a low dose of 1 x 10^3^ CAR T cells followed by LNP-RNA vaccination achieved greater CAR T cell levels 11 days after CAR T cell dosing than mice receiving a 1000-fold higher dose of 1 x 10^6^ CAR T cells but not the vaccine. Finally, the authors hypothesize that administration of a lower initial dose of CAR T cells followed by serial vaccination will lead to CAR T cell levels in the optimal therapeutic range while avoiding dose limiting toxicities observed by high dose administration and insufficient therapeutic activity observed when CAR T cells fail to persist (98).

#### 3.4.2 CAR engagement by antigen supplementation

Another approach to increase CAR engagement is to engineer cells to express the CAR-specific antigen and thus provide antigen supplementation (**Figure 1D**). Two studies have explored antigen supplementation to enhance HIV-specific CAR T cell persistence in the absence of HIV viremia (23, 99). Uninfected BLT humanized mice or ART-suppressed non-human primates (NHP), respectively, were administered a cell line, K562, engineered to express HIV Env. This led to proliferation of the HIV-specific CAR T cells in both models and prolonged time to viral rebound in the NHPs (23, 99). K562 cells have been explored extensively as artificial APC given their lack of HLA surface expression, ease of engineering, and good safety profile (100–102). Antigen supplementation by artificial APCs therefore also represents a potential “vaccination-like” strategy for expanding CAR T cells *in vivo* in the absence of high antigen levels.

#### 3.4.3 Engagement of the native T cell receptor

Most CAR T cells retain their native TCR which has been exploited in an effort to enhance the persistence of the CAR T cells (**Figure 1D**). Adoptive transfer of T cells with TCR-specificity toward chronic viral infections can persist when administered to recipients of allogeneic bone marrow transplant and reconstitute the cellular immunity toward these infections (103–105). By enriching virus-specific T cells these can be engineering to express a CAR directed against a different antigen while retaining the TCR specificity. The CAR T cells can then expand through engagement with the TCR-specific antigen in the absence of the CAR-specific antigen (106–111). Several pre-clinical studies have demonstrated improved expansion and persistence *in vivo* of CAR T cells engineered from virus-specific T cells when vaccination directed to the TCR is administered (112–114) Importantly, virus-specific CAR T cells does not expand when a vaccine directed to a different TCR-specificity is administered (112).

This indirect CAR T cell expansion approach has been explored in clinical trials using Epstein-Barr virus (EBV)-specific T cells engineered to express a CAR against a tumor antigen (115–118). In a clinical trial from 2008, Pule and colleagues (115) evaluated EBV-specific GD-2-CAR T cells in 11 participants. The EBV-specific GD-2-CAR T cells were detectable twice as long as GD-2-CAR T cells without EBV-TCR specificity. Additionally, CAR T cells sampled at different time points up to 6 months after infusion were cultured with autologous EBV-expressing B cells and the CAR transgene was quantified by qPCR. Only EBV-specific GD-2-CAR T cells expanded in response to EBV-TCR stimulation (115). In a 2019 clinical trial, Lapteva and colleagues (116) assessed EBV-specific CD19-CAR T cells. They found that the EBV-specific CD19-CAR T cells expanded and persisted in participants with reactivation of EBV leading to engagement of the native EBV-specific TCR (116). Lastly, Cruz and colleagues (117) performed a clinical trial assessing the safety of infusing virus-specific allogeneic CD19-CAR T cells to recipients of allogeneic HSPC transplantation. No graft-versus-host reaction was observed, which the authors hypothesized was due to the virus-specificity of the TCR. Two study participants had viral reactivation of EBV which coincided with an increase in the EBV-specific CD19-CAR T cells suggesting that EBV-TCR engagement induced an expansion of the EBV-specific CAR T cells (117). Lastly, Guan and colleagues (114) evaluated HIV-specific CAR T cells made from Cytomegalovirus (CMV)-specific T cells with CMV vaccination during HIV-suppressive ART. They observed that for the same dose of HIV CAR T cells, only the CAR T cells with CMV-specific TCR expanded after administration of a CMV vaccine (114).

Although TCR-engagement can lead to substantial CAR T cell expansion, TCR and CAR cross-talk have important implications for the functionality of CAR T cells (119). Yang and colleagues (120) demonstrated that CAR T cells produced from T cells with TCR specificity to the ubiquitously expressed male minor histocompatibility antigen HY have diverse *in vivo* activity in the presence of TCR- or CAR antigens (120). In the presence of TCR antigen, CD8+ CAR T cells did not expand and had diminished efficacy while CD4+ CAR T cells expanded and retained their effector functions (120). Furthermore, the indirect CAR T cell expansion strategy require the presence of the native TCR. Recent efforts in allogeneic CAR T cell development have targeted CAR integration to the TCR alpha constant locus (TRAC) (121). This results in disruption of the native TCR while driving the CAR from the endogenous TRAC promoter leading to greater antitumor activity (121). Disruption of the native TCR would thus preclude the indirect CAR T cell expansion method but may become an “off-the-shelf” CAR T cell product by bypassing the need for individual cell engineering.

## 4 Conclusion

Ensuring long-term persistence of HIV-specific CAR T cells is key to achieving durable control of the infection in the absence of ART. Here, we have described strategies to enhance the expansion and persistence of CAR T cells. We argue that successful CAR T cell trials in HIV will likely require combinations of the described approaches to ensure persistence of the CAR T cells and ultimately achieve a cure for HIV.

## Author contributions

FR: Conceptualization, Visualization, Writing – original draft, Writing – review & editing. NL: Visualization, Writing – review & editing. OS: Writing – review & editing. MT: Conceptualization, Funding acquisition, Supervision, Writing – review & editing.
